# CircRNA circFADS2 is under-expressed in sepsis and protects lung cells from LPS-induced apoptosis by downregulating miR-133a

**DOI:** 10.1186/s12950-022-00300-3

**Published:** 2022-03-12

**Authors:** Fang Niu, Xiaofeng Liang, Jindi Ni, Zhuye Xia, Lijing Jiang, Hong Wang, Hongjie Liu, Guofeng Shen, Xiang Li

**Affiliations:** 1 Department of Critical Care Medicine, Second Hospital of Lanzhou University, 730000 Lanzhou City, Gansu Province P. R. China; 2 Department of Infectious Diseases, Jiujiang Maternal & Child Health Care Hospital, 332000 Jiujiang City, Jiangxi Province P. R. China; 3 Department of Critical Care Medicine, Minhang Hospital, Fudan University, No. 39 Xinling Road, Minhang District, 201199 Shanghai, P. R. China; 4 Department of Cardiology, Minhang Hospital, Fudan University, 201199 Shanghai, P. R. China

**Keywords:** Sepsis, circFADS2, miR-133a, LPS, Apoptosis

## Abstract

**Background:**

It has been reported that hsa_circRNA_100833 (identified as circFADS2) and miR-133a play opposite roles in LPS-induced cell apoptosis, which contributes to the development of sepsis. This study was carried out to explore the interaction between circFADS2 and miR-133a in sepsis.

**Methods:**

Expression of circFADS2 and miR-133a in plasma from both sepsis patients (*n*=62) and healthy controls (*n*=62) was studied by RT-qPCR. Pearson’s correlation coefficient analysis was utilized to analyze the correlation between circFADS2 and miR-133a levels across plasma samples from sepsis patients. Cell viability and apoptosis, levels of proteins associated with apoptosis (cleaved caspase-3 and cleaved caspase-9), and expression of pro-inflammatory cytokines in LPS-treated HBEpCs were detected by MTT assay, cell apoptosis assay, western blot, and ELISA, respectively. In addition, a dual-luciferase reporter assay was performed to verify the interaction between circFADS2 and miR-133a.

**Results:**

CircFADS2 was under-expressed (0.56-fold vs. control) in sepsis, and miR-133a was highly expressed (2.05-fold vs. control) in sepsis. An inverse correlation between circFADS2 and miR-133a was observed across sepsis samples. LPS decreased cell viability, increased cell apoptosis, and elevated productions of tumor necrosis factor (TNF)-α, interleukins (IL)-1β, IL-6, and IL-8 in HBEpCs in a dose-dependent manner. In addition, circFADS2 was identified as a target gene of miR-133a. The further experiment revealed that circFADS2 overexpression and miR-133a inhibition prominently promoted cell viability (1.71-fold vs. pcDNA3.1; 1.65-fold vs. NC miRNA) and decreased apoptosis of LPS-treated HBEpCs (0.44-fold vs. pcDNA3.1; 0.47-fold vs. NC miRNA). Moreover, circFADS2 knockdown and miR-133a overexpression inhibited viability (0.36-fold vs. pcDNA3.1; 0.37-fold vs. NC miRNA) and increased apoptosis (1.54-fold vs. pcDNA3.1; 1.51-fold vs. NC miRNA) of LPS-treated HBEpCs. Notably, circFADS2 overexpression reduced the effects of miR-133a on LPS-treated HBEpCs.

**Conclusions:**

CircFADS2 is under-expressed in sepsis and may protect lung cells from LPS-induced apoptosis by downregulating miR-133a.

**Supplementary Information:**

The online version contains supplementary material available at 10.1186/s12950-022-00300-3.

## Background

Sepsis is a major challenge in clinics that is caused by the body’s responses to severe infections[1]. Sepsis is usually caused by infections of bacteria, viruses, and fungus, with bacterial infection as the major cause [2]. With proper treatment, such as systemic antibiotics within one hour of diagnosis, most patients with mild or moderate sepsis can get full recovery[3]. However, in severe cases, such as septic shock, organs failures may occur, leading to a mortality rate as high as 40% before hospitalization[4, 5]. Even worse, patients who recovered from severe sepsis are prone to future infections[6], resulting in an increased risk of death within 2 years after sepsis.

The failures of organs, such as the lung, liver, kidney, and heart, are the major causes of death among sepsis patients[7–9]. Therefore, the prevention and treatment of organ failures are the keys to the survival of sepsis patients. With the increased elucidation of the molecular mechanism of sepsis, several molecular players have been proven to be potential targets for the treatment of organ failures, such as acute lung injuries, in sepsis patients[10–12]. For example, Sirtuin 1 (SIRT1), a NAD^+^-dependent histone deacetylase and transcriptional enhancer of GR, has been found to restrain lung inflammasome activation in a murine model of sepsis[13]. Myeloid differentiation factor-2 (MD2), a binding protein of lipopolysaccharide (LPS), has been found to be essential to LPS recognition and the subsequent mediation of toll-like receptor 4 (TLR4)-dependent sepsis and acute lung injury [12]. Circular RN[10]As (circRNAs) are RNAs with loop structures that are generated by aberrant splicing [14]. Rather than coding RNAs, circRNAs are covalently closed non-coding RNAs that play critical roles in human diseases mainly by regulating gene expression[15, 16]. Therefore, circRNAs are potential targets for treating diseases. Recently, Li et al. revealed that circFADS2 protected LPS-treated chondrocytes from apoptosis by acting as an interceptor of miR-498/mTOR cross-talking [17]. It has been reported that circRNA circFADS2 and miR-133a play opposite roles in LPS-induced cell apoptosis[17, 18], which contributes to the development of sepsis[19]. Tacke et al. demonstrated that elevated miR-133a level is correlated with disease severity in sepsis and a predictor of mortality [20].

The present study was aimed to investigate the mechanism of circFADS2 in a LPS‑induced sepsis human bronchial epithelial cell (HBEpC) model. CircFADS2 overexpression and miR-133a inhibition reduced apoptosis and increased viability of LPS-induced HBEpCs. The regulatory effects of circFADS2 on sepsis may be achieved partly by targeting miR-133a. Identifying the role of circFADS2/miR-133a axis in sepsis may contribute to developing new targets for sepsis treatment.

## Methods

### Sepsis patients and healthy controls

A total of 62 sepsis patients (male/female: 32/30; mean age, 52.3±4.9 years) who were admitted to Minhang Hospital, Fudan University between March 2018 and March 2020 were enrolled in the study. In addition, 62 healthy controls (male/female: 32/30; mean age, 52.4±4.8 years) who were at the Physiological Health Center of the hospital for routine systemic physical examination were recruited in the study. All healthy controls had normal physical parameters. To exclude other factors that could affect gene expression, patients with initiated therapy, other clinical disorders, and history of sepsis were excluded. All sepsis cases were caused by bacterial infections and diagnosed by blood test to show the existence of bacteria. All patients and controls signed informed consent. The current study was approved by the Ethics Committee of the Minhang Hospital, Fudan University. Blood samples were taken from sepsis patients within 24 h of admission. The blood samples of healthy participants were acquired during their physical examination.

### Cell culture and LPS treatement

Blood samples (2 ml) were extracted from the elbow veins of both sepsis patients and healthy controls prior to therapy into tubes containing 0.2 ml of citric acids and centrifuged for 15 min at 1200 g to separate plasma.

Lung cells in this study were human bronchial epithelial cells (HBEpCs) from Sigma-Aldrich and cultured in bronchial Epithelial Cell Medium (Sigma-Aldrich) at 37 °C in an incubator with 5% CO_2_ incubator and 95% humidity to about 85% confluence prior to the subsequent assays. For LPS treatment, 1 × 10^6^ HBEpCs were cultured in media containing 0, 2, 4, 8, and 12 µg/ml LPS for 48 h.

### Knockdown assays

The small interfering RNAs (si-circFADS2 and si-NC) were synthesized and purchased from GenePharma (Shanghai, China). MiR-133a inhibitor and negative control (NC inhibitor) were purchased from Invitrogen. For knock-down assay, 1 × 10^6^ HBEpCs were transfected with 50 nM siRNA or miR-133a inhibitor using Lipofectamine 2000 (Invitrogen) following the manufacturer’s protocol.

### Overexpression assays

CircFADS2 expression vector and miR-133a mimicwere purchased from GenePharma and Sigma-Aldrich (USA), respectively. For overexpression assay, 1 × 10^6^ HBEpCs were transfected with 1 µg CircFADS2 expression vector or 50 nM miR-133a mimic using Lipofectamine 2000 (Invitrogen) following the manufacturer’s protocol. Cells were washed with fresh medium after incubation with transfection medium for 6 h and cultured in fresh medium for 48 h prior to subsequent analysis.

### RT-qPCR assays

Total RNAs in plasma and HBEpCs were extracted using Ribozol (VWR) and digested with a DNA eraser (Takara, Japan) until all samples reached an OD260/280 ratio close to 2.0, which indicated pure RNA. Electrophoresis (5% urea-PAGE gel) was carried out to analyze the integrity of RNA samples. Only RNA samples with high purity and satisfactory integrity were subjected to subsequent assays. The cDNA was synthesized using reverse transcription kit (Fermentas, USA). RT-qPCR assay was performed using ReverTra Ace™ qPCR RT Kit (Toyobo, Japan). GAPDH and U6 were selected as internal controls. Ct values of circFADS2 and miR-133a were normalized to their endogenous controls using the 2^-ΔΔCt^ method. The primer sequences were presented in Supplementary Table 1.

### Western blot

The whole cell proteins were extracted using RIPA lysis buffer (Gibco) and quantified by using a bicinchoninic acid protein assay kit (Pierce; Thermo Fisher Scientific, Inc.).

After quantification, protein sample was separated by 10% SDS-PAGE gels and transferred onto PVDF membranes (Millipore Sigma, Billerica, MA). Afterward, the membrane was blocked in 5% non‑fat milk for 2 h at room temperature and incubated with anti-human cleaved caspase-3 (ab2302, 1:1,000, Abcam) and cleaved caspase-9 (ab2324, 1:1,000, Abcam) overnight at 4 ℃. After the incubation with goat anti-rabbit second antibody (ab205718, 1:5,000, Abcam), the protein levels were detected by enhanced chemiluminescence substrate (ECL, Millipore Sigma) and quantified using Image Lab™ Software (Bio-Rad).

### MTT assay

Cells were grown in 96-well plates and underwent various treatments. On the next day, 10 µl of MTT (5 mg/ml, Sigma) was added to each well. After incubation for 4 h, cells were dissolved in 100 µl of dimethyl sulfoxide (DMSO, Sigma), and the absorbances at 570 nm were measured using a microplate reader (Molecular Devices, San Jose, CA) to determine cell viability.

### Cell apoptosis assay

The apoptosis ratio was analyzed using the Annexin V-FITC Apoptosis Detection Kit (Beyotime, China). In brief, HBEpCs were collected and transferred to 6-well cell culture plates with 8000 cells in 1.5 ml medium per well. After treatment, cells were digested with 0.25% trypsin-EDTA solution and then suspended by PBS. After being centrifuged at 1000 rpm for 5 min, 1 × 10^5^ cells were incubated with 5µL Annexin V-FITC (BD Biosciences, USA) for 15 min and 5µL propidium iodide for another 5 min. Apoptotic cells were detected by flow cytometer (BD Biosciences, USA). Data were analyzed using CellQuest analysis software (BD Biosciences, USA).

### ELISA

After different treatments, cell culture supernatants were collected, and the contents of interleukins tumor necrosis factor (TNF)-α (ab181421), IL-6 (ab178013), IL-8 (ab46032), and (IL)-1β were analyzed using ELISA kits (Abcam, Cambridge, UK) following the manufacturer’s instructions.

### Dual‑luciferase reporter assay

Dual‑luciferase reporter assay was performed to detect the interaction between circFADS2 and miR-133a. circFADS2 wild-type (circFADS2-WT) or circFADS2 mutated type (circFADS2-Mut) reporter vectors were synthesized by General Biosystems. 1 × 10^6^ HBEpCs were co‑transfected with circFADS2-WT (or circFADS2-Mut) and miR-133a (NC miRNA) using lipofectamine 3000. 48 h later, the luciferase activity was detected using the dual-lucy assay kit (Beijing Solarbio Science & Technology co., ltd.).

### Statistical analysis

Data were presented as the mean ± standard deviation. Comparisons were conducted with a Student’s t test (for 2 groups) or one-way ANVOA followed by a Tukey’s post hoc test (for ≥3 groups). Pearson’s correlation coefficient analysis was performed to analyze the correlations between circFADS2 and miR-133a. A *p*<0.05 value was considered statistically significant. Experiments were repeated three times independently.

## Results

### Altered expression of circFADS2 and miR-133a in plasma samples from sepsis patients

**Fig. 1:**
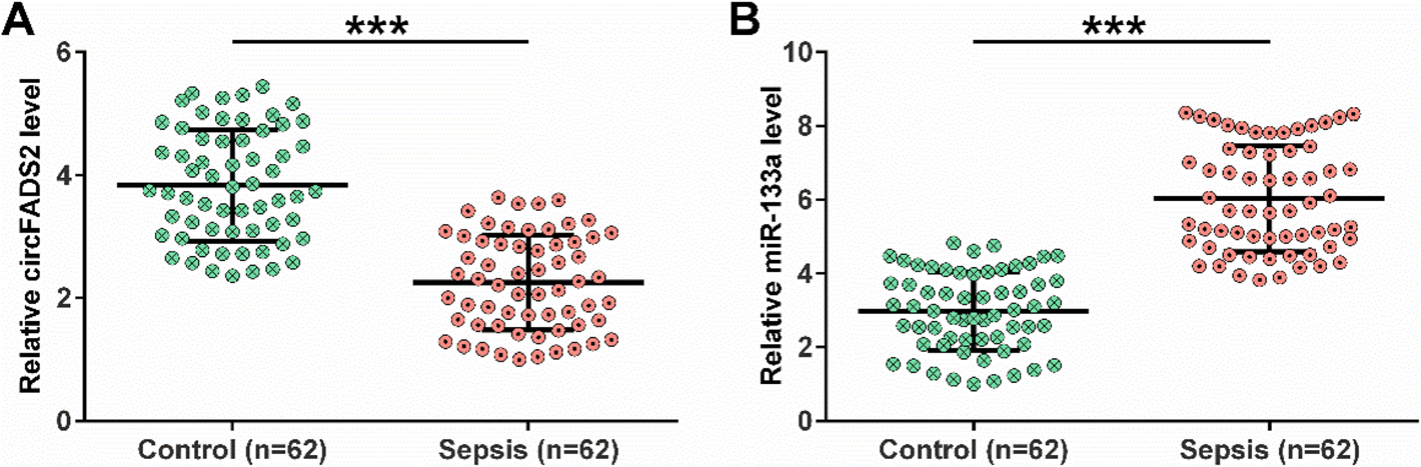
Sepsis patients showed altered expression of circFADS2 and miR-133a. RNA samples isolated from the plasma samples of sepsis patients (*n*=62) and healthy controls (*n*=62) were subjected to RT-qPCR to analyze the differential expression of circFADS2 (**A**) and miR-133a (**B**) in sepsis. Ct values were normalized to corresponding internal controls using the 2^−ΔΔCt^ method. The sample with the biggest ΔCt value was set to value “1” and used to normalize other samples to calculate relative gene expression. ***, *p*<0.001.

RNA samples isolated from the plasma samples of 62 sepsis patients and 62 healthy controls were subjected to RT-qPCR to analyze differential gene expression in sepsis. Our data revealed that compared to the 62 healthy controls, circFADS2 was under-expressed in sepsis (Fig. 1 A, *p*<0.001), and miR-133a was overexpressed (Fig. 1B, *p*<0.001) in sepsis. Therefore, circFADS2 downregulation and miR-133a overexpression may participate in sepsis.

### CircFADS2 and miR-133a levels in plasma samples from sepsis patients were inversely correlated

**Fig. 2:**
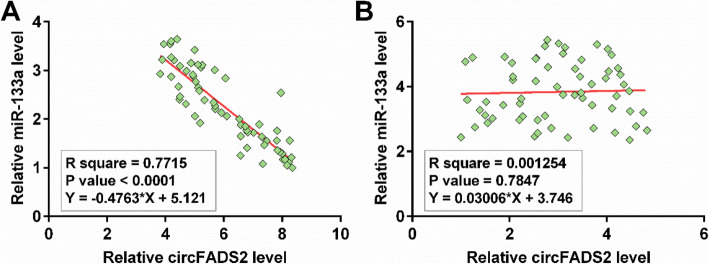
CircFADS2 and miR-133a levels in plasma samples from sepsis patients were inversely correlated. The differential expression pattern of circFADS2 and miR-133a in sepsis may indicate potential crosstalk between them. To explore their interaction, Pearson’s correlation coefficient analysis was performed to analyze the correlations between circFADS2 and miR-133a across sepsis samples (**A**) and control samples (**B**).

The differential expression pattern of circFADS2 and miR-133a in sepsis may indicate potential crosstalk between them. To explore their relationship, Pearson’s correlation coefficient analysis was performed. The results revealed that circFADS2 and miR-133a were inversely and significantly correlated across plasma samples from sepsis patients (Fig. 2 A), but not across plasma samples from healthy controls (Fig. 2B). Their close correlation indicated a potential interaction between them.

### LPS treatment altered the expression of circFADS2 and miR-133a in HBEpCs

**Fig. 3:**
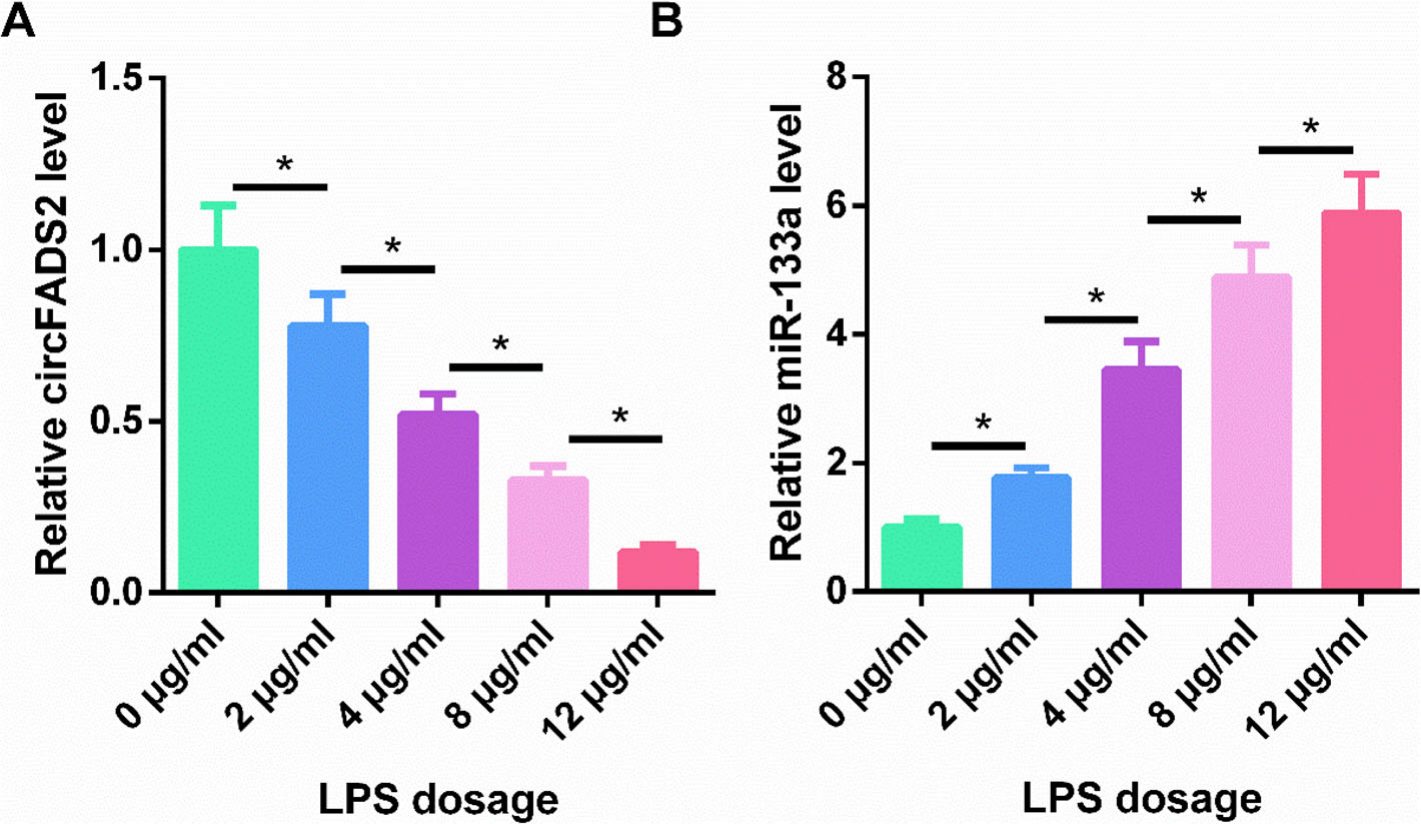
LPS treatment altered the expression of circFADS2 and miR-133a in HBEpCs. 1 × 10^6^ HBEpCs were treated with 0, 2, 4, 8, and 12 µg/ml LPS for 48 h, and the expression of circFADS2 (**A**) and miR-133a (**B**) were detected using RT-qPCR. *, *p*<0.05.

HBEpCs were treated with 0, 2, 4, 8, and 12 µg/ml LPS for 48 h, and the expression of circFADS2 and miR-133a were detected using RT-qPCR. It was observed that LPS treatment decreased circFADS2 expression (Fig. 3 A, *p*<0.05) and increased miR-133a expression in a dose-dependent manner (Fig. 3B, *p*<0.05).

### LPS induced HBEpC apoptosis and inflammation

**Fig. 4:**
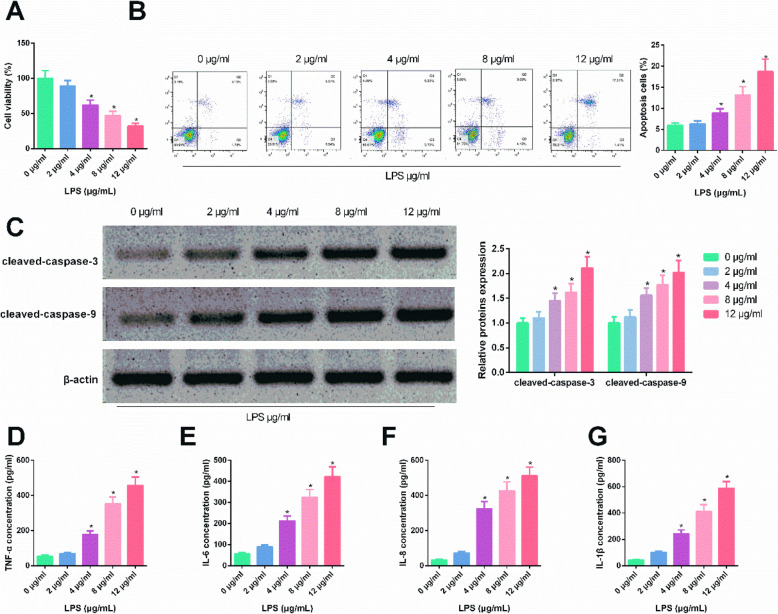
LPS induced HBEpC apoptosis and inflammation. 1 × 10^6^ HBEpCs were treated with 0, 2, 4, 8, and 12 µg/ml LPS for 48 h. Cell viability was detected using MTT assay(**A**). Cell apoptosis was measured using Annexin V-FITC apoptosis detection assay(**B**). Western blot was used to detect the protein levels of cleaved caspase-3 and cleaved caspase-9 (**C**). ELISA was performed to determine the concentrations of TNF- α, IL‑6, IL‑8, and IL‑1 β. *, *p*<0.05.

Cell biological function was assessed to verify the successful establishment of the sepsis cell model. Compared with controls, MTT assay results showed that LPS treatment significantly inhibited HBEpC viability in a dose-dependent manner (Fig. 4 A, *p*<0.05); Flow cytometry analysis indicated that LPS treatment significantly increased HBEpC apoptosis (Fig. 4B, *p*<0.05). Consistently, Western blot showed that LPS significantly enhanced the levels of cleaved caspase-3 and cleaved caspase-9 (Fig. 4 C, *p*<0.05). Moreover, ELISA analyses showed that LPS treatment significantly increased concentrations of TNF-α, IL‑6, IL-8, and IL-1 β in HBEpCs in a dose-dependent manner (Fig. 4D-G, *p*<0.05). These results suggested that LPS induced cell apoptosis and inflammation, confirming the successful establishment of the sepsis cell model.

### CircFADS2 directly targeted miR-133a

**Fig. 5:**
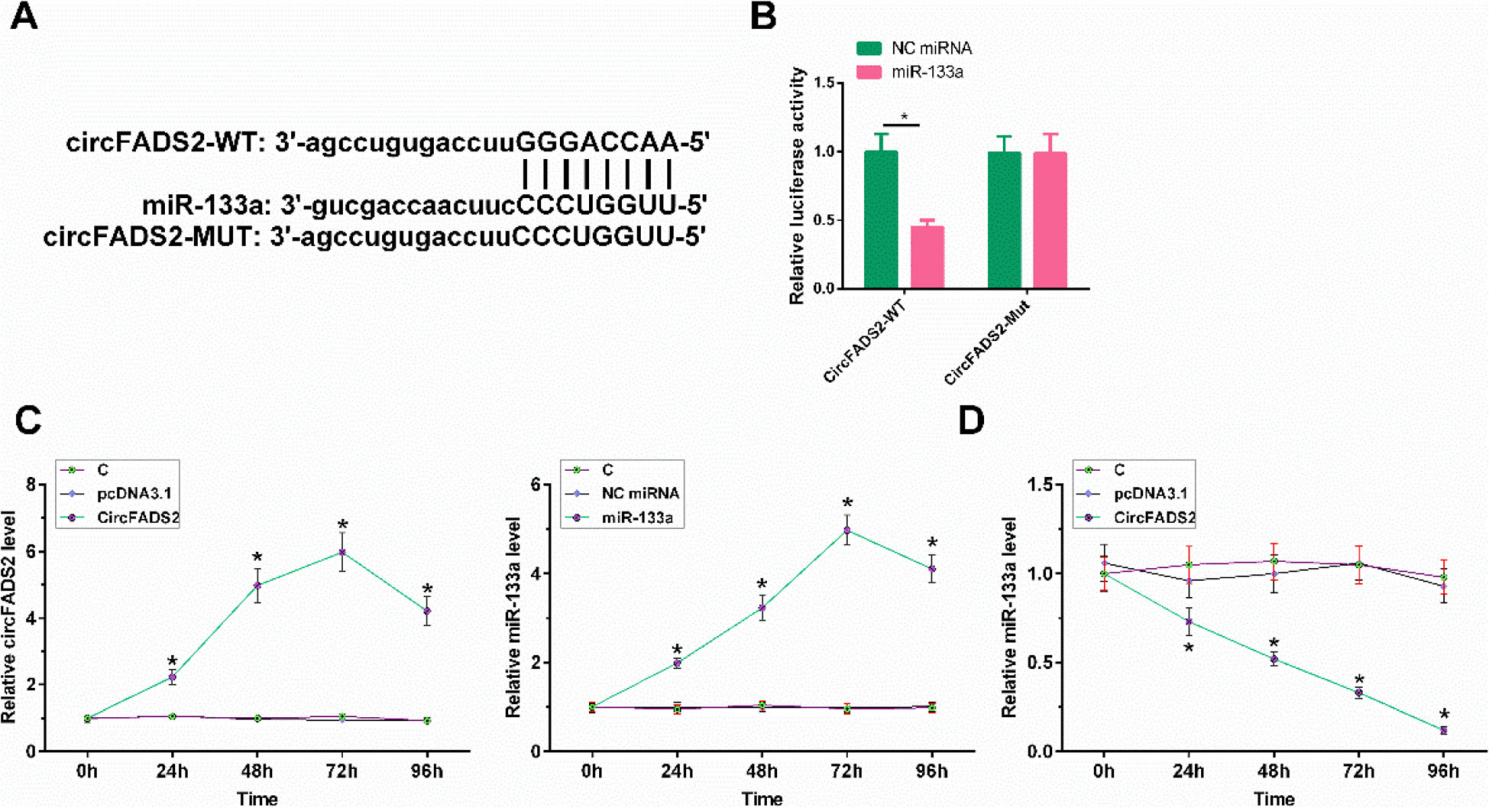
CircFADS2 directly targeted miR-133a. The putative binding sites between circFADS2 and miR-133a and circFADS2 MUT predicted by StarBase software (**A**). 1 × 10^6^ HBEpCs were co-transfected with circFADS2-WT (or circFADS2-Mut) and miR-NC (or miR-133a). 48 h later, the luciferase activity was detected by Dual‑luciferase reporter assay (**B**). 1 × 10^6^ HBEpCs were transfected with 1 µg CircFADS2 expression vector or 50nM miR-133a mimic. 48 h later, the expression levels of circFADS2 and miR-133a were detected by RT-qPCR(**C**). 1 × 10^6^ HBEpCs were transfected with 1 µg CircFADS2 expression vector. 48 h later, miR-133a level was detected by RT-qPCR (**D**). *, *p*<0.05.

Analysis utilizing StarBase revealed that circFADS2 was a sponge of miR-133a (Fig. 5 A, *p*<0.05). To validate this prediction, a dual-luciferase reporter assay was performed. As shown in Fig. 5B, co-transfection with miR-133a significantly inhibited the luciferase activity of circFADS2-WT compared with the NC miRNA group. There was no significant difference in luciferase activity in circFADS2-Mut. CircFADS2 overexpression and miR-133a overexpression were achieved in HBEpCs at 48 to 96 h of post-transfection with circFADS2 expression vector and miR-133a mimic, respectively (Fig. 5 C, *p*<0.05). Moreover, circFADS2 overexpression decreased miR-133a expression in HBEpCs (Fig. 5D, *p*<0.05).

### CircFADS2 overexpression inhibited apoptosis of HBEpCs induced by LPS via miR-133a

**Fig. 6:**
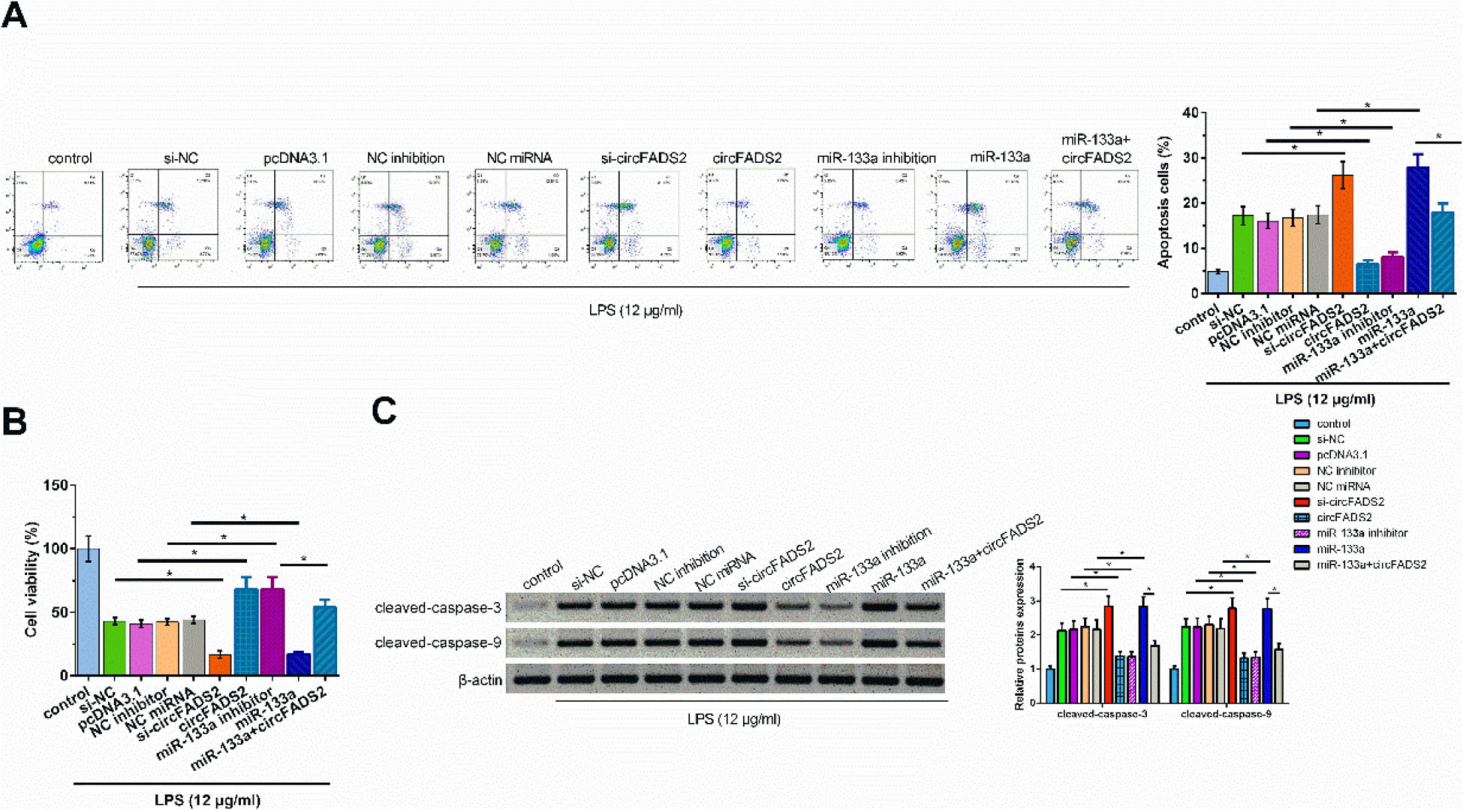
CircFADS2 overexpression inhibited LPS-induced HBEpCs apoptosis via miR-133a. 1 × 10^6^ HBEpCs were treated with 12 µg/ml LPS, and transfected with si-circFADS2, circFADS2 expression vector, miR-133a mimic or inhibitor. 48 h later, the apoptotic cells were detected by Annexin V-FITC Apoptosis detection assay (**A**). Cell viability was detected by MTT assay (**B**). The protein levels of cleaved caspase 3 and cleaved caspase 9 were detected by Western blot (**C**). *, *p*<0.05.

The effects of overexpression of circFADS2 and miR-133a on the apoptosis of HBEpCs induced by LPS were analyzed by cell apoptosis assay. Our data showed that circFADS2 overexpression and miR-133a knock-down inhibited apoptosis, while circFADS2 knockdown and miR-133a overexpression promoted cell apoptosis in LPS-induced HBEpCs (Fig. 6 A, *p*<0.05). In addition, circFADS2 overexpression reduced the stimulative effects of miR-133a overexpression on cell apoptosis induced by LPS (Fig. 6 A, *p*<0.05). MTT assay showed that circFADS2 overexpression and miR-133a inhibition promoted cell viability, while circFADS2 knockdown and miR-133a overexpression inhibited cell viability in LPS-induced HBEpCs (Fig. 6B, *p*<0.05). CircFADS2 overexpression neutralized the suppressive effects of miR-133a overexpression on cell viability in LPS-treated HBEpCs (Fig. 6B, *p*<0.05). Western blot revealed that circFADS2 knockdown and miR-133a overexpression increased the levels of cleaved caspase 3 and cleaved caspase 9, while circFADS2 overexpression and miR-133a inhibition had opposite effects on these two proteins (Fig. 6 C, *p*<0.05). Moreover, circFADS2 overexpression reduced the stimulative effects of miR-133a overexpression on protein levels (Fig. 6 C, *p*<0.05).

## Discussion

The involvement of circFADS2 and miR-133a in sepsis and their potential crosstalk in sepsis were explored in this study. We found that circFADS2 and miR-133a levels were altered in sepsis. Interestingly, circFADS2 and miR-133a played opposites roles in the apoptosis of HBEpCs induced by LPS, and circFADS2 might suppress cell apoptosis by downregulating miR-133a.

LPS is an endotoxin that is able to regulate the development of myocardial injury caused by sepsis and has been widely used to induce sepsis models *in vitro* [21]. In the present study, the effect of LPS on HBEpCs was measured. The data from RT-qPCR, MTT assay, cell apoptosis assay, and Western blot suggested that LPS treatment reinforced apoptosis and inflammatory responses and inhibited viability of HBEpCs, which was in line with previous studies [22], thus indicating the successful establishment of a sepsis model *in vitro*.

The functions of circFADS2 have been explored in cancer biology[23, 24]. In lung cancer and colorectal cancer, circFADS2 is overexpressed and promotes cancer progression by regulating cancer cell behaviors, such as increasing cell proliferation and invasion[23, 24]. Besides that, a recent study reported that circFADS2 protects chondrocytes from LPS-induced apoptosis via the miR-498/mTOR axis[17]. It is well known that LPS-induced cell inflammation are critical contributors to sepsis[19]. RT-qPCR in the present study revealed that circFADS2 was downregulated in sepsis and LPS-treated HBEpCs. Furthermore, the Annexin-V/PI apoptosis assay showed that circFADS2 siRNA transfection significantly promoted HBEpC apoptosis and inhibited HBEpC viability, while circFADS2 overexpression showed opposite effects on HBEpC apoptosis and viability. Western blot showed that circFADS2 siRNA significantly increased the levels of cleaved caspase 3 and cleaved caspase 9, while circFADS2 overexpression showed the opposite effects on these two apoptotic proteins. Taken together, circFADS2 might protect LPS-induced HBEpCs from apoptosis and enhance their viability.

It has been reported that miR-133a in sepsis can target SIRT1 to aggravate inflammation[18]. MiR-133a regulates inflammasome to promote myocardial injury by regulating NLRP3 in ischemic mouse hearts [25]. MiR-133a also functions as a tumor suppressor in prostate cancer [26], cervical cancer [27], and lung cancer [28]. In the present study, miR-133a was highly expressed in sepsis. LPS treatment increased the expression of miR-133a in a dose-dependent manner. In addition, miR-133a was predicted to be a target of circFADS2 by StarBase software. The dual-luciferase reporter assay revealed that miR-133a overexpression significantly inhibited the luciferase activity in cells transfected with wild-type circFADS2, suggesting that circFADS2 served as a miR-133a sponge. Moreover, a significant inverse correlation between circFADS2 and miR-133a was found in the plasma of sepsis patients. Furthermore, miR-133a inhibition promoted cell viability and inhibited cell apoptosis and miR-133a overexpression suppressed cell viability and accelerated cell apoptosis in LPS‑treated HBEpCs. Functional analyses demonstrated that circFADS2 overexpression reversed the effects of miR-133a on cell viability and cell apoptosis.

The present study has some limitations. *In vivo* animal model experiments are needed to further confirm the function of circFADS2 in sepsis. In addition, it’s worth noting that a significant inverse correlation between circFADS2, and miR-133a was revealed in plasma of sepsis patients, but not in the plasma of healthy controls. We suspected that in pathological conditions, some unknown pathological factors drive the altered expression of circFADS2/miR-133a axis, which deserves to be further studied. Hong et al. reported that circFADS2 is overexpressed in sepsis, and LPS treatment increased circFADS2 expression in a does-dependent manner[29]. In this study, we found circFADS2 is downregulated in sepsis, and LPS treatment inhibited circFADS2 expression in a does-dependent manner. We all found that circFADS2 is specifically expressed and plays an important role in sepsis, although the roles are different. This may be because circFADS2 regulates sepsis through different mechanisms, and the specific reasons will be explored in future studies.

## Conclusions

CircFADS2 is downregulated in sepsis, and circFADS2 overexpression may protect lung injuries in sepsis by reducing LPS-induced apoptosis via downregulating miR-133a.

## Supplementary information

**Additional file 1**

## Data Availability

The analyzed data sets generated during the present study are available from the corresponding author on reasonable request.
