# Supplementary material for: CircRNA circFADS2 is under-expressed in sepsis and protects lung cells from LPS-induced apoptosis by downregulating miR-133a
Source: J Inflamm (Lond). 2022 Mar 12;19:4. doi: 10.1186/s12950-022-00300-3 (PMC8917694; doi:10.1186/s12950-022-00300-3)
Supplement: Supplementary file 1 — Additional file 1 [file 12950_2022_300_MOESM1_ESM.docx]

Supplementary Table 1. The Sequences of Primers Used in RT-PCR

| cDNA | Primer sequences |
| --- | --- |
| circFADS2 (divergent) |  |
| F | 5′-GCCAACTGGTGGAAT-3′ |
| R | 5′-GTGCAGCATGTTCAC-3′ |
| circFADS2 (convergent) |  |
| F | 5’-GAAGGCGATTGGATCAGAATC-3’ |
| R | 5’-TAACACCGGCCATGCAGATCT-3’ |
| GAPDH |  |
| F | 5′-GTGGCCGAGGACTTT G-3′ |
| R | 5′-CCTGTAACAACGCAT CT-3′ |
| miR-133a-3p |  |
| F | 5′ -UUUGGUCCCCUUCAACCAGCUG-3′ |
| R | 5’ -UAAACCAAGGUAAAAUGGUCGA-3’ |
| U6 |  |
| F | 5’ -CGCTTCGGCAGCACATATAC-3’ |
| R | 5’ -TTCACGAATTTGCGTGTCAT-3’ |
